# Retrolaparoscopic adrenalectomy assisted by three-dimensional reconstructed digital model in a patient with situs inversus totalis

**DOI:** 10.1186/s12957-018-1473-8

**Published:** 2018-08-20

**Authors:** Xiaobin Yuan, Bin Zhang, Caoyang Hu, Xuhui Zhang, Dongwen Wang

**Affiliations:** Department of Urology, First Hospital of Shanxi Medical University, No. 85, Jiefangnan Road, Taiyuan, 030001 Shanxi China

**Keywords:** Situs inversus totalis, Adrenalectomy, Retrolaparoscopic surgery, Adrenal adenoma, Digital model

## Abstract

**Background:**

Situs inversus totalis is a relatively rare congenital anomaly. Performing the retrolaparoscopic adrenalectomy for the patient with situs inversus totalis is a skill-demanding and challenging surgical task, which has been even more rarely reported.

**Case presentation:**

We present a case with a large right adrenal mass (10.2 × 9.4 × 7.9 cm) complicated by situs inversus totalis. This 59-year-old female patient underwent the retrolaparoscopic adrenalectomy in our department. In order to facilitate the surgical orientation and improve the manipulating accuracy, the data from computed tomography images was extracted and the three-dimensional digital model was reconstructed. Under the assistance of preoperative planning and intraoperative navigation by the three-dimensional digital model, the retrolaparoscopic adrenalectomy was technically precise and successful. The targeted adrenal tumor was excised completely with final pathological diagnosis of adrenocortical adenoma.

**Conclusions:**

Retrolaparoscopic adrenalectomy can be performed safely in patients with situs inversus totalis. The assistance of preoperative planning and intraoperative navigation by the reconstructed three-dimensional digital model can facilitate the operation and lead to more precise vessel manipulation and accurate excision of tumor that is both effective and safe.

**Electronic supplementary material:**

The online version of this article (10.1186/s12957-018-1473-8) contains supplementary material, which is available to authorized users.

## Background

Situs inversus totalis (SIT) is a relatively rare congenital anomaly characterized by the complete mirror-imaged visceral organs in the opposite anatomic positions [1], and its incidence was reported to be in the range of 1:10000 to 1:20000 [2]. There are only four published papers that described the laparoscopic adrenalectomy for adrenal mass in the patients with SIT [3–6], but they were all finished via the transperitoneal approach. Here, we present a rare case of a much larger adrenal tumor (10.2 × 9.4 × 7.9 cm) with SIT who underwent the retrolaparoscopic adrenalectomy. To the best of our knowledge, our case is the first report that received the laparoscopic adrenalectomy via retroperitoneal way instead of transperitoneal way, and has the biggest reported adrenal tumor mass in SIT patient by far.

## Case presentation

A 59-year-old female patient was admitted into our hospital for the abnormal computed tomography (CT) image presentation of right adrenal mass revealed by occasional health examination, accompanied with the complaint of intermittent nausea and blurred vision. She had been diagnosed with SIT when she received the hysterectomy and near-total thyroidectomy for the uterus myoma and thyroid cancer in 2000. Besides, her past medical history also included the diabetes mellitus (type 2) for 6 years and significant hypertension for 5 years, ranging from 150 to 200/90 to 102 mmHg, fluctuated periodically.

The patient’s height was 158.0 cm and the body weight was 70.0 kg. The BMI was 28.04 kg/m^2^, which was well correlated with her significant abdominal obesity. The laboratory tests presented the normal levels of the serum potassium concentration (4.2 mEq/L) and the renin activity (3.8 ng/mL/h). The plasma cortisol and aldosterone (217 pg/mL) concentrations were also within the reference range. Besides, there were no abnormalities found in the levels of the urinary catecholamines (59.2 mcg/24 h) and vanillylmandelic (4.3 mg/24 h) either.

**Fig. 1:**
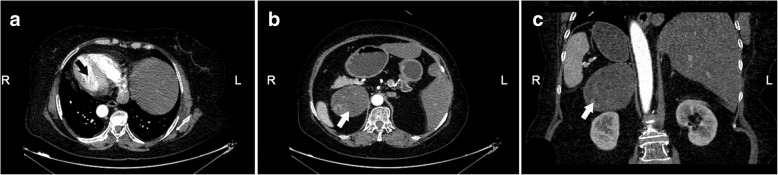
The CT findings. Situs inversus totalis was confirmed, and a mass of about 10 cm in diameter in contact with the right adrenal gland was detected. **a** Dextrocardia (black arrow: cardiac apex). **b** The axial plane (white arrow: the targeted adrenal mass). **c** The coronal plane (white arrow: the targeted adrenal mass)

As for the medical image examinations, the routine chest X-ray image revealed the dextrocardia; the dual-source 64-slice enhanced CT (LightSpeed VCT, GE Healthcare, USA) scan including arterial phase, venous phase, and excretory phase after intravenous contrast administration confirmed the diagnosis of SIT and presented her round-shaped mass on the right adrenal gland with the size of 10.2 × 9.4 × 7.9 cm (Fig. 1).

**Fig. 2:**
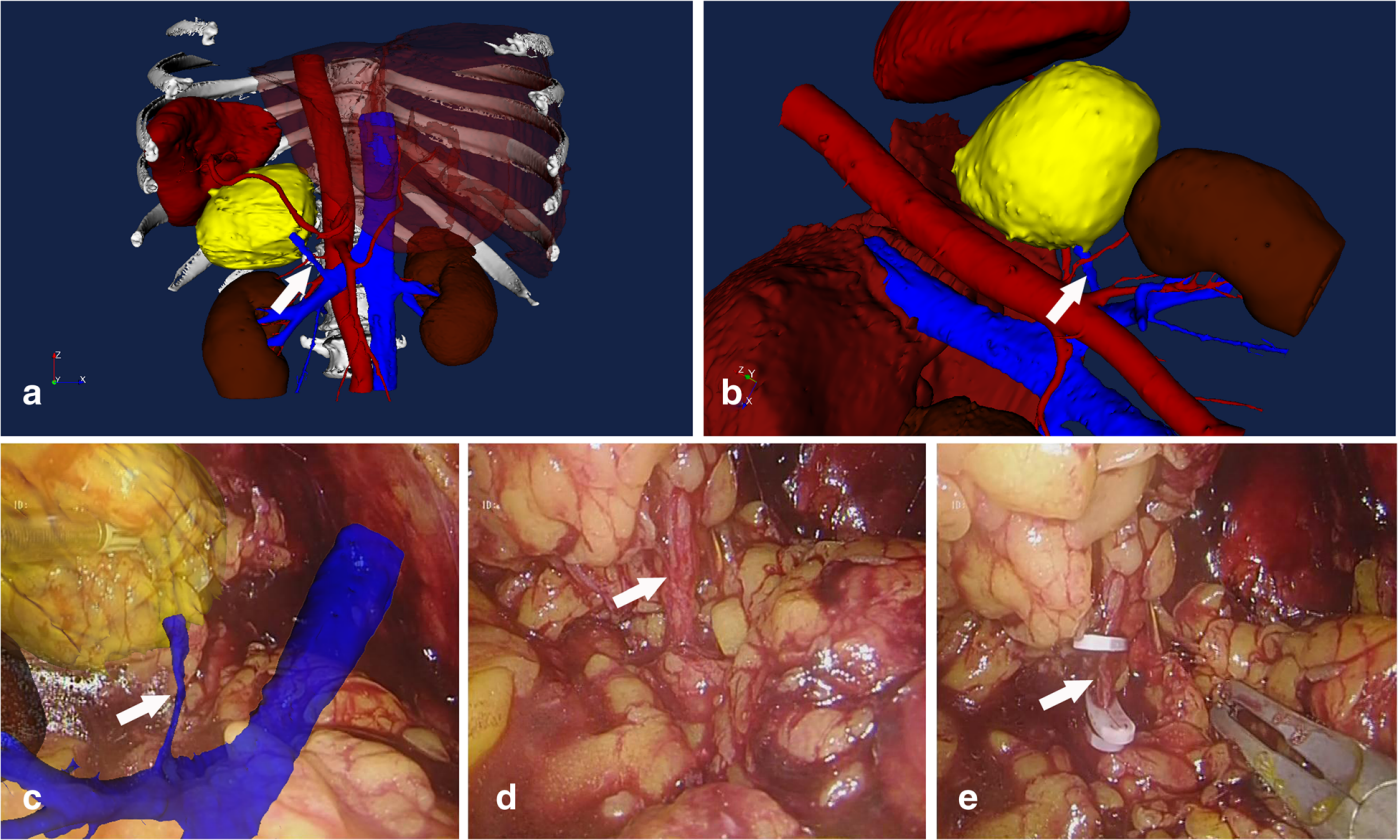
**a**, **b** The three-dimensional digital model (3D-DM) was reconstructed using different colors (white arrows: the central vein of adrenal gland). **c** The intraoperative fused vasculature featured the targeted central vein of adrenal gland (the white arrow). **d**, **e** Identifying and clamping the central vein of adrenal gland (the white arrows)

In order to facilitate the surgical orientation and improve the manipulating accuracy, the data from CT images was extracted and the three-dimensional digital model (3D-DM) was reconstructed: The original image data from CT scanning was set in the format of Digital Imaging and Communications in Medicine (DICOM). The copied information was analyzed and reconstructed into the 3D-DM by using a postprocessing software named three-dimensional medical image reconstructing and guiding system (3D-MIRGS, China), which is a multifunctional workstation for the clinical application, whose functions included CT-based image reconstruction, preoperative planning, and intraoperative-assisted navigation [7]. The retroperitoneal space along with critical anatomic structures including adrenal tumor, the relevant vasculature, the kidney, and the renal collecting system on the affected side were reconstructed and marked by using different colors simultaneously (Fig. 2).

The morphometric calculation and analysis of the reconstructed 3D-DM provided the surgeons with the valuable anatomic information such as the spatial locations of vital vessels, maximal diameter, and margins of the adrenal mass. The tumor can be shadowed and turned into a transparency, leaving the hollow-shaped crater. A more distinct spatial relationship between the adrenal mass and the nearby structures showed clearly via this intraparenchymal visualization. Based on these data, we tailored a specific surgical plan for this rare case. The surgeons can gain a full comprehension of the regional complexity which is mirrored to the normal anatomy.

The retrolaparoscopic adrenalectomy was performed at the Department of Urology. The patient was placed on the operating table in the lateral decubitus position with the affected side upward. The general anesthesia and tracheal intubation were administrated. After padding the pressure points with beanbags and fixing the posture with the optimal table flexion, a longitudinal 1.5 cm incision for 12 mm trocar was made in the posterior axillary line below the 12th rib. In order to minimize the potential irritation to the stability of blood pressure, the retroperitoneal space was dilated in the blunt finger-dissecting style instead of the traditional ballooning way. Another two 12 mm trocars were located at the point of 2 cm above from iliac crest superior border in the mid-axillary line for the laparoscope, and at the point under the subcostal margin in the anterior axillary line for laparoscopic instruments respectively. After the routine insertion of three trocars and the establishment of pneumoperitoneum at a pressure of 10 mmHg, the retroperitoneal fat tissue was removed under the laparoscopic surveillance. Another assisted 5 mm trocar was inserted due to the patient’s obesity. A well-trained full-time surgical technician captured some typical screenshots to illustrate the anatomical landmarks. Then, the semitransparent 3D-DM were superimposed onto these screenshots with appropriate axis and size adjustments. The composite 3D-DM images provided the surgeon with information relative to the inverted anatomy, thereby acting as a kind of assisted navigation for the subsequent manipulations. All these speedy manual image fusions performed synchronously during operation and all the fused images were displayed on a separate screen. Under the assisted navigation of 3D-DM, the Gerota fascia was incised and the adrenal central vein was dissected carefully, following its ligation by using 5 mm Hem-o-lok clips. After that, the mass was excised completely with the careful preservation of normal adrenal tissue and the other adrenal vessels including the adrenal arteries were ligated and scissored up. Then, the retroperitoneal pressure was set down to the level of 5 mmHg and the hemostasis was achieved carefully. Given the size of the tumor mass and the skin elasticity, we extended the incision below the 12th rib along its axis for the total length about 7 cm. The specimen of adrenal tumor was packed into a homemade laparoscopic pouch with a string by graspers and then was withdrawn by holding and pulling the string vigorously. The trocar incisions were carefully closed, and a rubber drainage catheter was left in situ.

**Fig. 3:**
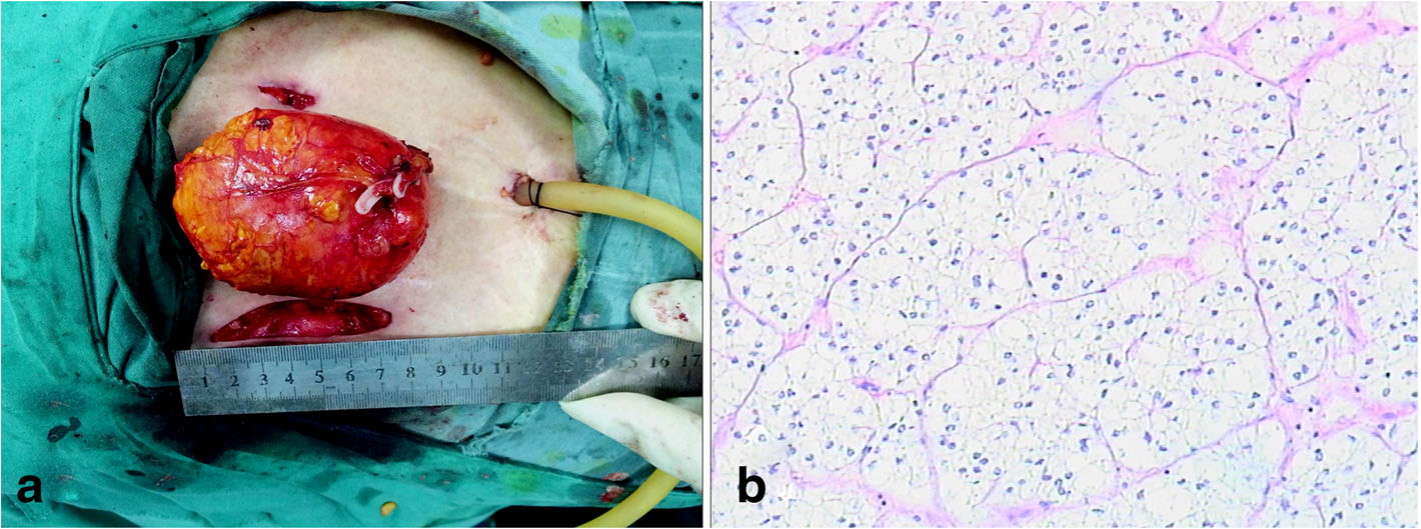
**a** The size of resected tumor was 10.0 × 9.1 × 6.8 cm, and no gross extracapsular invasion was found. **b** The final pathological diagnosis was adrenocortical adenoma

The operative time was 1 h and 10 min, with no intraoperative complications happened. The estimated blood loss was about 10 mL. The size of resected tumor was 10.0 × 9.1 × 6.8 cm, and no gross extracapsular invasion was found. The final pathological diagnosis was adrenocortical adenoma (Fig. 3). The postoperative course was uneventful, and the patient was discharged after 4 days postoperatively. A video demonstrating the operation accompanies this article (Additional file 1).

## Discussion

After more than two-decade global verification via massive clinical practice, laparoscopic adrenalectomy has been viewed as the gold standard [8] of surgical treatment for adrenal masses since the first report by Gagner et al. [9] in 1992. Many reports have proven the effectiveness and safety of transabdominal laparoscopic adrenalectomy and its well tolerance to a broad spectrum of functioning and non-functioning adrenal diseases [10, 11]. The motivations for less irritation to the bowel function and faster postoperative recovery, together with the improvements in endo-surgical techniques and experiences, have supported the feasibility and safety of retrolaparoscopic way for adrenalectomy, with no exception to this challenging task of endoscopic resection to the large adrenal tumor complicated with SIT.

To the dextromanual surgeons, the mirror-imaged anatomy of SIT patient always indicates the technical challenges in endoscopic orientation and maneuvers. The inefficient familiarity to the abnormal disposition of organs and the consequent reduced self-confidence in the surgical manipulations will lead to more cautious and repeated spatial confirmations, which can result in the prolonged operative course inevitably [12, 13]. Besides, the mirror-imaged anatomy also requires to rearrange the positionings of the surgeons and the surgical devices to facilitate the operation.

To ensure a safe and smooth surgical procedure, accurate preoperative anatomic comprehension and adequate preoperative planning work are useful and pivotal. In this context, our surgeon team held a routine session to formulate a suitable surgical blueprint for this case based on the information gained from the detailed morphometry of the reconstructed 3D-DM preoperatively. The surgical plan consists of four parts: the planning of acquainting with the mirror-imaged retroperitoneal space, the planning of confirming and dissecting the central vein of the adrenal gland, the planning of accurate adrenal mass excision with maximal preservation of the normal adrenal tissue, and the planning of avoiding damage to the inverted proximal anatomical structures. The most accurate information about the mirror-imaged regional anatomy featuring the valuable warning landmarks where potential surgical injury may occur will be noticed and emphasized. It has been proven to be helpful and could achieve a more ample preparation by walking through the surgical procedure in this style prior to the operation.

Compared with the conventional CT scanned images, 3D-DM helped the surgeon to generate an improved and more detailed surgical blueprint in his mind, which undoubtedly increased his comprehension and confidence on the upcoming operation and eventually resulted in the reducing of the tentative maneuvers in the vessel-controlling and tissue-dissecting procedures. Moreover, when it came to many surgical decision-making occasions in the operation, the rapid feedback according to the information provided by 3D-DM shortened the evaluating time efficiently on the premise of safety.

Nevertheless, we still recommend that SIT-involved retrolaparoscopic adrenalectomy should be performed by the experienced laparoscopic surgeon, as the intraoperative fast and suitable solution to the SIT-initiated spatial orientation problems still mostly rely on the professional attainment and surgical skills of the surgeon.

In our future clinical practice, we believe the robotic retrolaparoscopic adrenalectomy might be helpful to these rare cases. The centered robotic view of the surgical field can eliminate the difficulty of changing instruments in both hands [14].

## Conclusions

Retrolaparoscopic adrenalectomy can be performed safely in patients with SIT. The assistance of preoperative planning and intraoperative navigation by the reconstructed 3D-DM can facilitate the operation and lead to more precise vessel manipulation and accurate excision of tumor that is both effective and safe.

## Additional file

Additional file 1: The 3D-DM guided preoperative planning and the intraoperative video. (WMV 57228 kb)

## Abbreviations

3D-DM: Three-dimensional digital model
CT: Computed tomography
DICOM: Digital Imaging and Communications in Medicine
SIT: Situs inversus totalis
